# A Set of Engineered *Escherichia coli* Expression Strains for Selective Isotope and Reactivity Labeling of Amino Acid Side Chains and Flavin Cofactors

**DOI:** 10.1371/journal.pone.0079006

**Published:** 2013-11-01

**Authors:** Jennifer Mehlhorn, Helena Steinocher, Sebastian Beck, John T. M. Kennis, Peter Hegemann, Tilo Mathes

**Affiliations:** 1 Institut für Biologie/Experimentelle Biophysik, Humboldt-Universität zu Berlin, Berlin, Germany; 2 Institut für Chemie, Humboldt-Universität zu Berlin, Berlin, Germany; 3 Biophysics Group, Department of Physics and Astronomy, Faculty of Sciences, Vrije Universiteit, Amsterdam, The Netherlands; Universidad de Granada, Spain

## Abstract

Biological reactions are facilitated by delicate molecular interactions between proteins, cofactors and substrates. To study and understand their dynamic interactions researchers have to take great care not to influence or distort the object of study. As a non-invasive alternative to a site-directed mutagenesis approach, selective isotope labeling in combination with vibrational spectroscopy may be employed to directly identify structural transitions in wild type proteins. Here we present a set of customized *Escherichia coli* expression strains, suitable for replacing both the flavin cofactor and/or selective amino acids with isotope enriched or chemically modified substrates. For flavin labeling we report optimized auxotrophic strains with significantly enhanced flavin uptake properties. Labeled protein biosynthesis using these strains was achieved in optimized cultivation procedures using high cell density fermentation. Finally, we demonstrate how this approach is used for a clear assignment of vibrational spectroscopic difference signals of apoprotein and cofactor of a flavin containing photoreceptor of the BLUF (Blue Light receptors Using FAD) family.

## Introduction

Since the advent of cloning in the early 1970s heterologous overexpression of proteins has become the method of choice for studying protein function on a molecular level. Especially structure-function relationships and molecular mechanisms of enzymes and sensory receptors can only be sufficiently addressed by studying the corresponding proteins *in vitro*. Methods that offer the necessary molecular resolution for this task like X-ray crystallography or NMR spectroscopy require large amounts of mono-disperse protein in high purity but are mainly suitable to describe the static molecular arrangement of the protein backbone and amino acid side chains. Although NMR is able to visualize the dynamic fluctuations of proteins down to the picosecond time domain [1,2], light- or chemical induced reactions that occur on a sub millisecond timescale like (photo-)sensory processes or single enzymatic turnovers may not be followed in real time, while vibrational spectroscopy can be performed in real time with femtosecond resolution instead [3,4]. Indeed FTIR and Raman spectroscopy have been key tools to elucidate the reaction mechanism of bacteriorhodopsin and rhodopsin [5]. Infrared difference spectroscopy is sensitive to changes in molecular vibrations and thereby provides information on the transformation of the inter- and intramolecular bond structure of a given biomolecule [5,6]. This information however is usually hard to extract for complex molecules like proteins, since vibrational modes of backbone, side chains and cofactor naturally overlap and are therefore difficult to assign and interpret. A clear assignment is experimentally accessible by selective heavy isotope labeling and thereby changing the frequency of the corresponding vibration. Compared to site-directed mutagenesis isotope labeling is a non-invasive protein modification and allows the clear identification and assignment of vibrational signatures by comparison of spectroscopic data from labeled and unlabeled variants. In this regard the labeling efficiency is especially important for vibrational spectroscopy, since contributions of labeled and unlabeled proteins are recorded at the same time.

A nice example for the usefulness of selective isotope labeling is the BLUF (Blue light receptors using FAD) photoreceptor family. BLUF photoreceptor domains are about 130 amino acid large proteins that non-covalently bind flavin as a blue light absorbing chromophore. These domains occur as single proteins or fused to enzymes involved in second messenger metabolism and regulate various physiological responses and life style decisions accordingly [7,8]. After excitation of the flavin with blue light the flavin binding pocket undergoes a subtle hydrogen bond switch, which results in a red shift of the visible absorbance of the flavin and a downshift of the flavin carbonyl signature [9,10]. The hydrogen bond switch occurs in less then one nanosecond after ultrafast proton coupled electron transfer between the flavin and a conserved tyrosine [11–15]. The molecular nature of the hydrogen bond switched state is still under considerable discussion, since X-ray and NMR structures are discussed conversely in terms of their assignment to light- or dark-adapted states [16–21]. All molecular schemes for the hydrogen bond switched state involve a conserved glutamine side chain, which may either rotate or tautomerize to facilitate a new hydrogen bond to the flavin [12,22–26]. Since no functional mutation of the glutamine side chain is possible and the vibrational signatures of flavin carbonyl groups and glutamine amide side chain are expected to overlap, a direct proof of either model may only be obtained by vibrational spectroscopy on selectively flavin or amino acid side chain isotope labeled BLUF domains.

For both IR and NMR spectroscopy global labeling approaches have been long established [27]. Heterologous expression systems like bacteria, yeast or insect cells are cultivated in defined media, which are composed of isotope enriched nitrogen and carbon sources for protein expression. Thereby complete labeling of all corresponding nuclei in the protein - and cofactor, if provided through biosynthesis by the expression host - is achieved. In many cases, however, a more selective labeling is desired, in order to reduce the amount of couplings in NMR or to specifically assign a signal in an IR difference spectrum [27,28]. A common way to achieve a reduced, but controlled isotope labeling is to feed specific, but non-uniform labeled carbon sources, which lead to a preferential isotope enrichment in certain amino acids [29–31], or to employ other carbon breakdown and *de novo* amino acid synthesis pathways [32]. In *Escherichia coli* it is also possible to selectively feed isotope labeled amino acids, which are quantitatively imported into the cell and incorporated into an overexpressed protein. Terminal amino acids (histidine e.g.), which do not serve as intermediates for other amino acid biosynthesis pathways and are not likely to cause undesired scrambling of isotopes, are especially suitable in this approach [33]. Furthermore, isotope scrambling and dilution can be suppressed by providing substrates that lead to feedback inhibition of chosen biochemical pathways or by application of selective inhibitors [28,34]. Most of these methods require extensive optimization of the cultivation conditions due to the complexity of the therefore required media. Alternatively, an efficient and convenient protein labeling environment free of isotope scrambling may be obtained by genomic disruption of the corresponding biochemical pathways [27,35]. 

**Figure 1:**
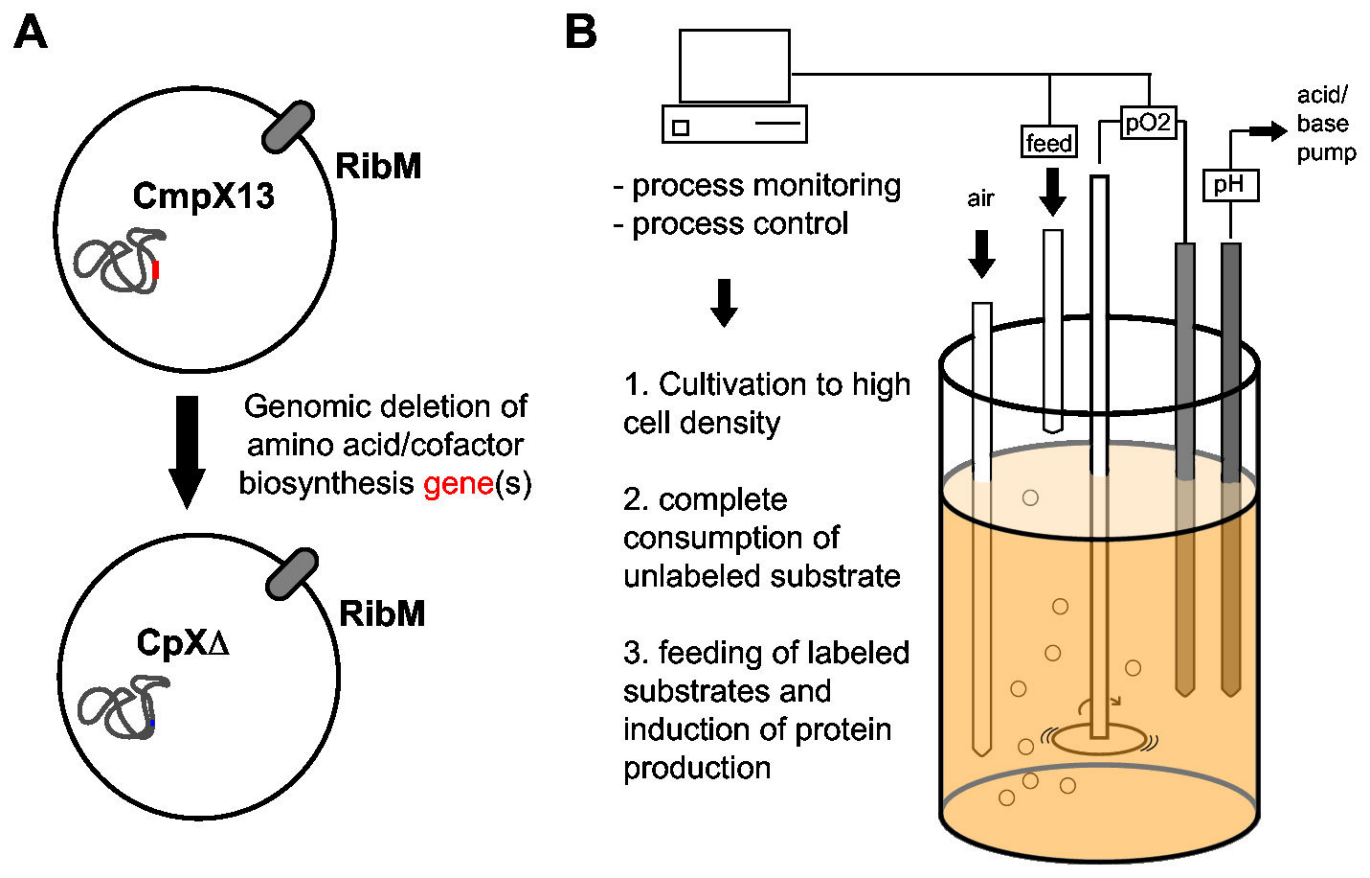
Amino acid and cofactor specific isotope labeling using custom-made auxotrophic expression strains (A) in a high cell density fermentation setup (B). CmpX13 is rendered auxotrophic for selected amino acid and/or cofactor synthesis pathways. The resulting expression strains are cultivated under controlled conditions to achieve the highest cell density under complete consumption of unlabeled substrates. Subsequently labeled substrates are fed and protein production is induced.

Here, we describe a straight-forward approach for selective labeling of amino acid side chains and flavin cofactors of a heterologously expressed protein. This strategy employs the disruption of amino acid and flavin biosynthesis pathways in an *E. coli* expression strain using standard homologous recombination techniques (Figure 1A) [36]. Similar approaches have been reported earlier, in which either well known auxotrophic *E. coli* mutants were transformed into suitable expression strains [27] or well known expression strains were rendered auxotrophic [35]. Here we use the previously engineered expression strain CmpX13 [37] as a parent strain. It contains a constitutively expressed riboflavin uptake system, which makes it especially suitable for strong overexpression of flavoproteins. Furthermore we created enhanced riboflavin auxotrophic strains in analogy to the previously described strain CmpX131 [37], but with additional expression of flavokinases to lower the riboflavin supplementation requirement. These strains facilitate efficient *in vivo* flavin reconstitution, which is especially useful for proteins that cannot be classically reconstituted *in vitro* by unfolding and refolding in the presence of the cofactor analog [38,39]. Additionally, we describe how these strains can be used for highly efficient selective isotope labeling in a high cell density fermentation (HCDF) procedure (Figure 1B) [40]. Finally, we illustrate the potential of this approach by presenting FTIR light-minus-dark difference spectra of the *Synechocystis* BLUF photoreceptor Slr1694 (SyPixD) with selective flavin and protein labeling patterns.

## Experimental Procedures

### Genomic modification of CmpX13

Genomic modifications were carried out using λ-Red homologous recombination according to Datsenko&Wanner’s approach [41] using improved recombinase providing plasmids [42]. PAGE purified oligonucleotides for linear DNA preparation by PCR (Reprofast polymerase, Genaxxon) were ordered from Sigma Genosys (Table 1). For recombination steps the bacteria were supplied with the λ-recombinase by heat induction of pSIM6 at 42°C for 10 minutes at OD_600_ ~ 0.4 /cm. Subsequently cells were harvested and washed twice with 10% glycerol (w/v) for transformation. Linear DNA (ca. 500 ng purified dsDNA) was introduced by electroporation at 1500 V, 150 Ω and 50 µF. 

**Table 1:** Oligonucleotides used for genomic modifications of *E. coli*.

**Name**	**Sequence 5’->3’**
CAT-5’	TTTGAATTCGACGTTGATCGGCACGTAAG	
CAT-3’	TTTGGATCCTTCCTTAGCTCCTGAAAATC	
ribF-5’	TTTGGATCCATGAAGCTGATACGCGGCAT	
ribF-3’	TTTAAGCTTTTAAGCCGGTTTTGTTAGCC	
FMN1sp-5’	TTTGGATCCATGACAGTTAATTTAGAAGA	
FMN1sp-3’	TTTAAGCTTATACCTTGAAAAATGGG	
catribF-5’	CTGCTATTGTGCTGAACAGAATGTCTTAACTGATTTCAGGAGTTGTAAGTACGTTGATCGGCACGTAAGA	
catribF-3’	TGGCTCGCTAAAGGCTATTCTATCGCCCCCTCTCCGGGGGCGATTTCAGATTAAGCCGGTTTTGTTAGCC	
catfmn1-3’	TGGCTCGCTAAAGGCTATTCTATCGCCCCCTCTCCGGGGGCGATTTCAGATTATACCTTGAAAAATGGGT	
DRFSA-5’	CTGCTATTGTGCTGAACAGAATGTCTTAACTGATTTCAGGAGTTGTAAGTGTACCGGATCCGTCGACCTG	
DRFSA-3’	TGGCTCGCTAAAGGCTATTCTATCGCCCCCTCTCCGGGGGCGATTTCAGAGGAATTCCCCGGGGGATCCG	
DtrpA-5’	GACATCTTCACCGTTCACGATATTTTGAAAGCACGAGGGGAAATCTGATGGTGTAGGCTGGAGCTGCTTC	
DtrpA-3’	TGCCGCCAGCGGAACTGGCGGCTGTGGGATTAACTGCGCGTCGCCGCTTTCGGCTGACATGGGAATTAGC	
DtyrA-5’	GGATCTGAACGGGCAGCTGACGGCTCGCGTGGCTTAAGAGGTTTATTATGGTGTAGGCTGGAGCTGCTTC	
DtyrA-3’	GATGATGTGAATCATCCGGCACTGGATTATTACTGGCGATTGTCATTCGCCGGCTGACATGGGAATTAGC	
DhisD-5’	AGTTCAATTCTGGTCCTGCCGATTGAGAAGATGATGGAGTGATCGCCATGGTGTAGGCTGGAGCTGCTTC	
DhisD-3’	ACGCGCTAAATCGGTAATAGTCACGGTGCTCATGCTTGCTCCTTAAGGGCCGGCTGACATGGGAATTAGC	
DglnA-5’	AGATTTCGTTACCACGACGACCATGACCAATCCAGGAGAGTTAAAGTATGGTGTAGGCTGGAGCTGCTTC	
DglnA-3’	GCGAAAAGTTTCCACGGCAACTAAAACACTTAGACGCTGTAGTACAGCTCCGGCTGACATGGGAATTAGC	
DasnA-5’	GGTTTTTGTTGCTTAATCATAAGCAACAGGACGCAGGAGTATAAAAAATGCGGCTGACATGGGAATTAGC	
DasnA-3’	CCTGCTCAGACGCTGGCGGCGATAAATTATTACAGCAGAGAAGGGACGCTTGTAGGCTGGAGCTGCTTCG	
DasnB-5’	ACAAGCAAACACAACAAGCAACAAATACCAGGTTAACGGAGAAGGTTATGCGGCTGACATGGGAATTAGC	
DasnB-3’	CGGATTTCACCGGGGCTGTTTCGCATTTCTTACTTATACGCCGACTGGTGTGTAGGCTGGAGCTGCTTCG	
DcysE-5’	GCCCGCGCAGAACGGGCCGGTCATTATCTCATCGTGTGGAGTAAGCAATGGTGTAGGCTGGAGCTGCTTC	
DcysE-3’	TACATCGCATCCGGCACGATCACAGGACATTAGATCCCATCCCCATACTCCGGCTGACATGGGAATTAGC	
DargA-5’	AGAATAAAAATACACTAATTTCGAATAATCATGCAAAGAGGTGTGCCGTGGTGTAGGCTGGAGCTGCTTC	
DargA-3’	CGCATGTCGCATCCGACGATTTTCATCGCTTACCCTAAATCCGCCATCAACGGCTGACATGGGAATTAGC	

A constitutive expression cassette was constructed by cloning the *cat* promoter, amplified from pKD3 [41] using the primers CAT-3’/-5’, inside the multiple cloning site of pUC18 using *Eco*RI and *Bam*HI. The resulting plasmid is henceforth named pUC18-*cat*. The *E. coli* flavokinase gene *ribF* was amplified from genomic DNA of CmpX13 using the primers ribF-5’/-3’ and cloned into pUC18-*cat* using *Hind*III/*Bam*HI, henceforth named pUC18-*cat*-*ribF*. The *S. pombe* flavokinase gene *fmn1* was amplified from an expression plasmid (kindly provided by Markus Fischer, University of Hamburg) [43] using the primers FMN1sp-5’/-3’ and cloned into pUC18-*cat* using *Hind*III/*Bam*HI, henceforth named pUC18-*cat*-*fmn1*.

For replacement of the riboflavin synthase gene *ribC*, a linear DNA fragment encoding a *neoR*-*sacB* selection/counter selection cassette was amplified by PCR from a *Bam*HI fragment of pUM-24 [44] using the primers DRFSA-5'/-3', which contain a 50 bp large 5' overhang homologous to the flanking regions of *ribC*. Recombinant clones were selected on kanamycin containing LB-RF-agar plates (50 µg/mL kanamycin, 50 µM riboflavin) and colony purified. The selection/counter selection cassette in a single, correctly identified clone was replaced using the same procedure by a linear fragment amplified from pUC18-*cat-ribF* and pUC18-*cat–fmn1* using the primers catribF-5’/-3’ and catribF-5’/catfmn1-3’, respectively (Table 1). Recombinant clones were then selected for sucrose tolerance on LB-agar plates containing 10% sucrose. Sucrose tolerant clones were confirmed by colony PCR and DNA sequence analysis. One confirmed clone of each transformation, henceforth-named CpXribF or CpXFMN, respectively, was used in the experiments described below. 

In strain CmpX13, the essential genes for amino acid synthesis have been deleted by homologous integration of a linear fragment of a *neoR*-cassette (amplified by PCR from pKD4 with the corresponding primer pairs D<gene*>*-5’/-3’) containing up- and downstream FRT-sites with flanking homology regions to the corresponding gene (Table 1). Recombinant clones were selected on kanamycin containing LB agar plates (50 µg/mL kanamycin) and confirmed by colony PCR. To remove the *neoR*-cassette one of the confirmed recombinants was supplied with FLP-recombinase by thermal induction using pCP20. After 6 hrs at 42°C the cells were plated on LB and selected for loss of kanamycin resistance. The resulting clones were analyzed by colony PCR and phenotype and confirmed by sequence analysis of PCR products from genomic DNA. The resulting strains were named as listed in Table 2.

**Table 2:** Expression strains and their properties.

**Strain**	**Genotype**	**Phenotype**	**Approx. aminoacid/glucose ratio (w/w) for optimal growth**
CmpX13	C41(DE3) *manX::ribM*	-	-
CmpX131	CmpX13 *ribC*-	RF-	-
CpXribF	CmpX13 *ribC::cat-ribF*	RF-	-
CpXFMN	CmpX13 *ribC::cat-fmn1*	RF-	-
CpXΔQ	CmpX13 *glnA-*	Q-	1
CpXΔQ*	CmpX13 *glnA-, asnB-*	Q-	1
CpXΔC	CmpX13 *cysE-*	C-	0.065
CpXΔW	CmpX13 *trpA-*	W-	0.004
CpXΔY	CmpX13 *tyrA-*	Y-	0.0026
CpXΔH	CmpX13 *hisD-*	H-	0.00025
CpXΔN	CmpX13 *asnA-,asnB-*	N-	0.04
CpXΔR	CmpX13 *argA-*	R-	0.5
CpXFΔQ	CpXribF *glnA-*	RF-; Q-	1
CpXFΔQ*	CpXribF *glnA-, asnB-*	RF-; Q-	1
CpXFΔW	CpXribF *trpA-*	RF-; W-	0.015

### Growth assays

Growth tests were carried out in M9 minimal medium in 96-well plates in a shaking incubator (Infinite M200 Pro, Tecan) at 37°C for up to 16 hours. The amount of cells was estimated by measuring the optical density at 600 nm. Each well was inoculated to an OD_600nm_ ~ 0.05 /cm from a pre-culture grown overnight in Luria Bertani medium. Before inoculation the cells were washed twice with equal volumes of M9 medium without amino acid or flavin supplementation. For the amino acid auxotrophic strains the M9 medium employed here only contained growth-limiting amounts of glucose (0.1-0.2% w/v) and the indicated amount of amino acid supplementation. In this way the ratio of amino acid/glucose consumption was determined (Table 2) and used accordingly in subsequent experiments.

### High cell density fermentation (HCDF)

HCDF was carried out in double concentrated M9 minimal medium using a computer controlled fermentation system equipped with two 500 ml fermentation vessels (Multifors, Infors AG, Bottmingen-Basel, Switzerland). The pH of the medium was monitored using pH glass electrodes (405-DPAS-SC-K8S, Mettler Toledo, Gießen, Germany) and continuously adjusted to 7.2 during fermentation using 2 M NaOH. The fermentation vessels were inoculated to OD_600nm_ ~ 0.5-0.8 /cm and cultivation was carried out at 37°C. Stirring speed was adjusted between 250 and 800 rpm according to the relative pO_2_ level in the medium, which was continuously monitored (InPro 6820/12/220, Mettler Toledo, Gießen, Germany). If the pO_2_ dropped below 30% the stirrer was gradually increased, otherwise it was gradually lowered. The end of the biomass production phase was determined automatically by a sudden rise in pO_2_. The complete consumption of glucose and ammonia was confirmed using quick test strips (Quantofix Ammonium/Glucose, Macherey&Nagel, Düren, Germany). After 30 minutes of pO_2_ > 60% the protein production phase was initiated by lowering the temperature to 25°C. After 10 minutes at T=25°C new substrates were started to be pumped into the fermentation vessel. Protein production was induced by 1 mM IPTG (final concentration) added another 10 minutes later. After 14 hours the temperature was lowered to 18°C and the cells were harvested subsequently. For isotope labeling L-tyrosine-phenyl-^13^C_6_ (99% ^13^C; Cortecnet, Voisins-Le-Bretonneux, France) and u-^13^C6 D(+)-glucose (99% ^13^C; Euriso-Top GmbH, Saarbruecken, Germany) were used.

### Protein purification

Heterologously expressed proteins were prepared according to previously published procedures [45].

### FTIR spectroscopy

Protein samples in H_2_O were concentrated to an OD_441nm_ ~70-100 /cm. 2-5 µl were placed between two CaF_2_ plates without spacer and sealed with silicon grease for tightness. FTIR spectra between 1800 and 1000 cm^-1^ were recorded using a Bruker IFS66s spectrometer with 3 cm^-1^ resolution. Light-minus-dark difference spectra were generated by recording 100 scans of background without application of blue light and 100 scans with application of blue light (LED Luxeon, 1W, 460 nm). To estimate experimental drifting during the measurements 100 scans of background and 100 scans of sample were recorded without application of blue light to generate a dark-minus-dark difference spectrum. 10 experiments of each light-minus-dark and dark-minus-dark spectra were averaged and the resulting datasets were subtracted to correct for experimental drift. The unprocessed spectra and their standard deviation are given in Figure S3.

### Mass spectrometry

For mass spectrometry, protein samples (~10 µg) were separated on a standard Laemmli SDS-PAGE [46]. Protein bands were excised and digested with trypsin as previously described [47]. Samples after in-gel trypsin digestion were dissolved in 10 µl 50% methanol, 0.1% formic acid and mixed with an equal volume of a 3 mg/ml solution of α-cyano-4-hydroxycinnamic acid in a solution of 84% acetonitrile, 13% ethanol and 0.003% trifluoroacetic acid. The mixtures were spotted onto the MALDI sample holder and measured in a MALDI Orbitrap XL (Thermo Scientific) with a spectral resolution of 60’000. The labeling degree was assessed by the peak intensities of unlabeled and labeled peptides.

## Results and Discussion

### Up-regulation of intracellular flavokinase activity lowers the riboflavin requirement of riboflavin auxotrophic *E. coli* strains

**Figure 2:**
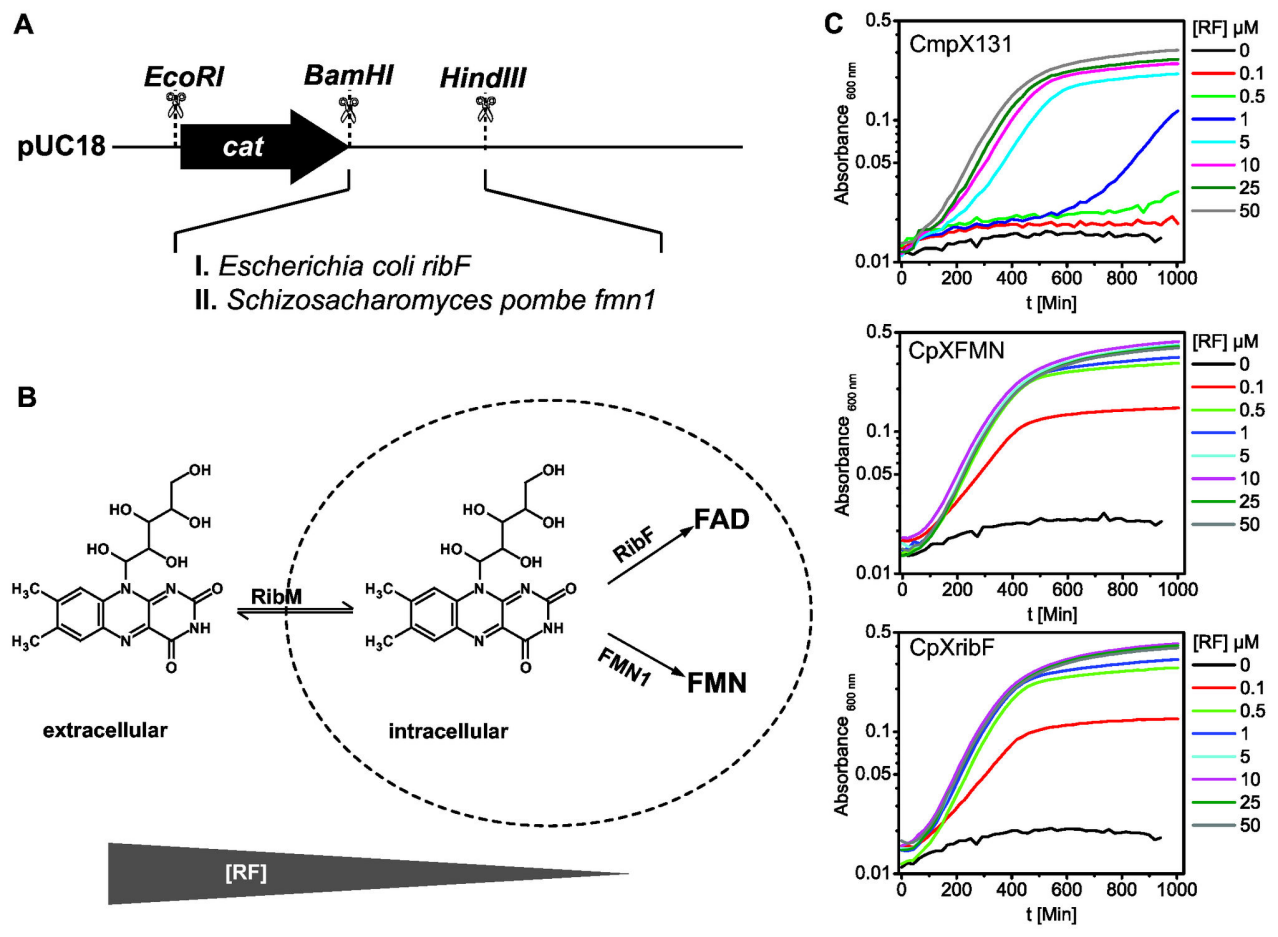
Cassette for constitutive expression of flavokinases in *E. coli* (A). The enhanced flavokinase activity of the strains CpXFMN and CpXribF containing the respective expression cassette reduces the intracellular amount of free riboflavin by accumulation of FAD and FMN, respectively. Thereby the transport equilibrium is shifted towards a higher uptake (B). CpXFMN and CpXribF accordingly show an enhanced growth at riboflavin concentrations below 10 µM as compared to the riboflavin auxotroph CmpX131 (C).

Even though the expression of the riboflavin transporter RibM lowers the riboflavin requirement of the riboflavin auxotrophic strain CmpX131 [37] significantly compared to other reported riboflavin auxotrophic mutants [48,49], further reduction of the riboflavin requirements is desirable for efficient *in vivo* isotope labeling or chemical modification of the flavin chromophore. Since RibM is a passive riboflavin uptake facilitator [50], a straight-forward way to increase the riboflavin flux into the cell is to shift the transport equilibrium by increasing intracellular flavokinase activity. Since RibM only transports riboflavin, phosphorylation and/or adenylation of riboflavin shifts the transport equilibrium in favor of riboflavin uptake. For this purpose we engineered a constitutive flavokinase expression cassette using the flavokinase either from *E. coli* (*ribF*) or from *Schizosaccharomyces pombe* (*FMN1*) under the control of the cat-promoter (Figure 2A). This cassette was introduced into the genome of CmpX13 to replace the gene for the riboflavin synthetase *ribC*. The flavin requirement of the resulting strains CpXribF and CpXFMN was estimated by growth tests in minimal medium with varying concentrations of riboflavin. Compared to the previously reported strain CmpX131 both flavokinase expressing strains show significantly enhanced growth at concentrations below 10 µM riboflavin and show growth limitations starting at 1 µM (Figure 2C). The growth curves of CmpX131 clearly show a delayed growth at riboflavin concentrations lower than 50 µM. In contrast, both CpXribF and CpXFMN1 may be cultivated equally efficient even at riboflavin concentrations down to 1 µM. The elevated flavokinase activity in these strains probably leads to an increase of cellular FMN and FAD concentrations in the case of the bifunctional *E. coli* flavokinase/adenylase RibF and FMN in the case of the monofunctional *S. pombe* flavokinase FMN1 (Figure 2B). We therefore suggest using CpXribF and CpXFMN for flavin labeling or flavin analog reconstitution experiments *in vivo*. The latter is expected to be similarly possible as with CmpX131, since all strains employ the same riboflavin transporter RibM. 

### A set of amino acid auxotrophic expressions strains: the CpX collection

Although various amino acid auxotrophic strains are available in many labs and culture collections (e.g. Keio collection [51]), we specifically chose the strain CmpX13, an expression strain which constitutively expresses a riboflavin transporter [37], for our experiments. Quantitative isotope labeling requires culture media composed of well-defined carbon/nitrogen sources, essential vitamins and trace elements. Since these so-called minimal media are less optimal for cell vitality as complex media like for example Luria Bertani (LB) broth, slow growth and low protein yield is usually observed. Additionally, the synthesis of cofactor precursors like riboflavin may be impaired and lead to a low cofactor availability during the overexpression of a given flavoprotein. With the ability of riboflavin uptake provided by the parent strain CmpX13, however, riboflavin can be supplied in the culture medium and thereby support endogenous flavin biosynthesis.

Selected amino acid synthesis genes were targeted and removed in CmpX13 using λ-Red mediated homologous recombination. The here presented modifications were selected to study various aspects in flavoprotein research in general and in BLUF photoreceptors in particular. While tyrosine and tryptophan are involved in electron transfer reactions in many flavo-enzymes and flavin photoreceptors, glutamine and asparagine were selected since they are integral parts of the hydrogen bond network between flavin and protein in BLUF photoreceptors. A histidine (H72 in Slr1694) is furthermore putatively involved in a hydrogen bond network with an aspartate residue (D69 in Slr1694), which undergoes changes in the protonation state in the dark recovery of BLUF photoreceptors [52]. Its particular role in the photoactivation process, however, is still unknown since mutation of either residue (D69 or H72) has not been accomplished yet. Arginine is of general interest for flavoproteins, since most FMN and FAD cofactors are bound non-covalently by one or more arginine residue via their phosphoester groups. The cysteine mutant was created for seleno-cysteine/-methionine labeling of flavoproteins. 

**Figure 3:**
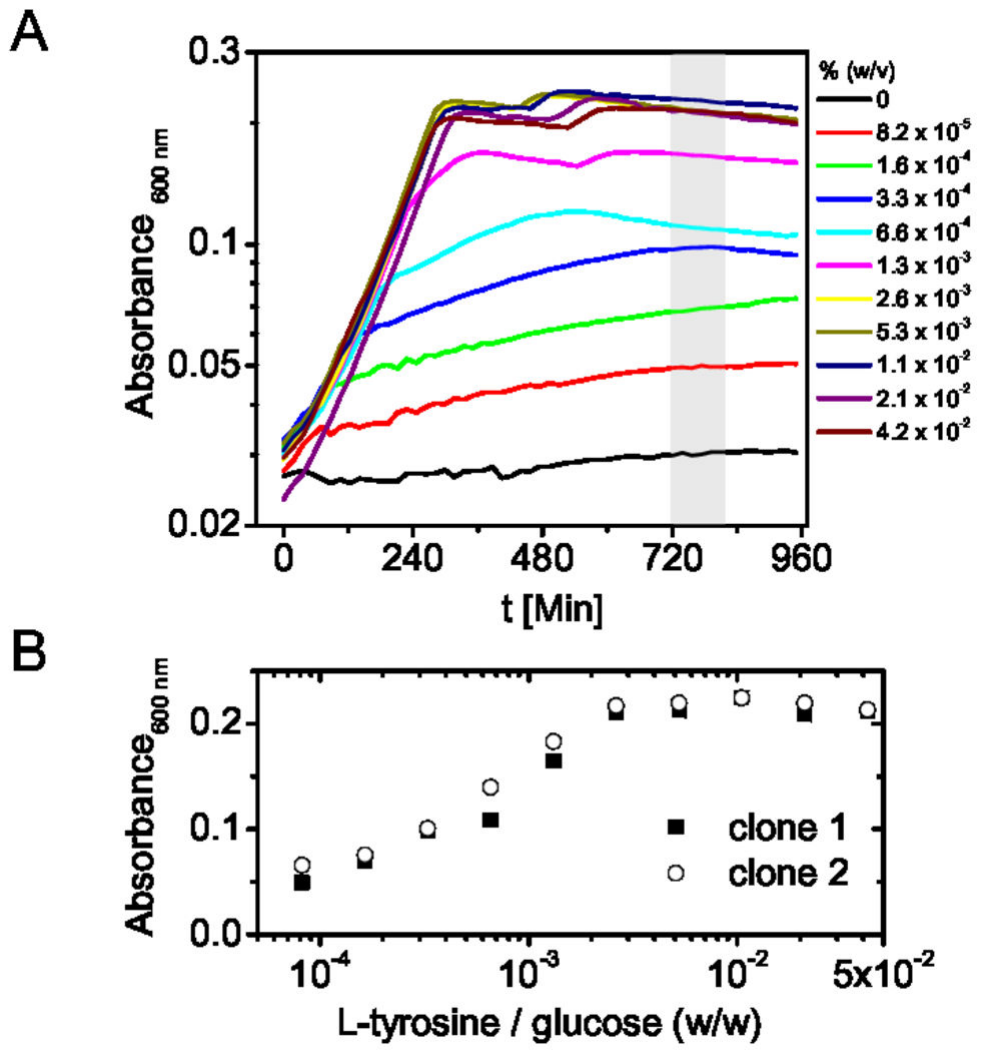
Tyrosine requirement of CpXΔY. Cells were grown in minimal medium supplied with the indicated amount of L-tyrosine. The cell density was estimated by monitoring the optical density at 600 nm at the indicated times (A). Cell densities in the stationary phase for two different clones were plotted against the tyrosine concentration (B). Thereby the relative amount of amino acid substrate to glucose for maximum cell density may be determined.

In contrast to all other strains described here the glutamine auxotrophic strains (Table 2) showed significant growth limitation on LB plates compared to wild type *E. coli* (not shown). Accordingly, these strains require high amounts of glutamine relative to the carbon source for optimal growth in minimal medium (Table 2). In M9 medium without amino acid supplementation all strains were unable to grow (Figure 3, Figure S1). While most of the here presented strains are single gene knockouts, the asparagine auxotrophic strain CpXΔN required two genes, *asnA* and *asnB*, to be removed for a complete asparagine auxotrophic phenotype. *asnB* encodes for a glutamine dependent asparagine synthase and may lead to nitrogen isotope dilution through unlabeled *de novo* synthesized glutamine. Since this enzyme may also lead to isotope scrambling from glutamine into asparagine in the glutamine auxotrophic strain CpXΔQ, we additionally removed *asnB* in the modified glutamine auxotrophic strain CpXΔQ*. Both glutamine auxotrophic strains however show virtually identically glutamine requirements, which might indicate that this pathway is rather insignificant for nitrogen isotope scrambling between asparagine and glutamine in these strains (Figure S2, Table 2). However, we did not further investigate in this direction here. Since the vitality of the double *glnA*-, *asnB*- mutant CpXΔQ* is apparently not affected by the additional mutation, we suggest using CpXΔQ* for further experiments.

In some cases a single cofactor-specific or amino acid-specific labeling may not be sufficient for a clear assignment and a selective double labeling of cofactor and amino acids is needed. This may be useful for downshifting selected flavin and amino acid signatures simultaneously to visualize underlying signals from other parts of the protein, but also for NMR techniques employing selective heteronuclear coupling to determine distances between flavin and amino acid side chains as well as their mutual orientations [53]. For this purpose we introduced auxotrophies for selected amino acids into the enhanced riboflavin auxotrophic strain CpXribF (see above). On top of their riboflavin auxotrophy the strains CpXFΔQ, CpXFΔQ* and CpXFΔW are additionally devoid of glutamine and tryptophan biosynthesis, respectively. The growth characteristics of these strains were compared with their corresponding single amino acid auxotrophs CpXΔQ, CpXΔQ* and CpXΔW (Figure S2, Table 2). Under conditions non-limiting for riboflavin, no significant difference between the riboflavin auxotrophs and prototrophs was found for the glutamine auxotrophic strains. However, the riboflavin/tryptophan double auxotroph CpXFΔW needs about three times more tryptophan for optimal viability than the single tryptophan auxotroph CpXΔW. The necessary amount however is still in the lower milligram range per gram of glucose. Therefore, these strains may be employed in a similar manner in respect to the amino acid requirement and may be used for simultaneous labeling of the flavin cofactor.

### High cell density fermentation of CpX strains for selective labeling of overexpressed proteins

Isotope labeled substrates are usually expensive and it is therefore of high interest to minimize the amount of substrate needed for protein labeling and to maximize protein yield. Cultivation of *E. coli* can be significantly improved by fermentation techniques, which provide optimal aeration and maintain a constant pH. Additionally fed-batch procedures, in which fresh carbon and nitrogen sources are added during the cultivation, may be employed to overcome nutrient limitations and optimize cell densities. In our approach we use a small volume high cell density fermentation procedure (HCDF), which automatically feeds the labeled substrate after the unlabeled substrate is consumed. In this way highest cell densities are achieved using unlabeled substrates in a biomass production phase and isotope labeled substrates are used exclusively in the protein production phase. For isotope labeling through the carbon or nitrogen source complete consumption of the unlabeled substrate is indicated by a sudden rise of the pO_2_ level in the culture, since no aerobic metabolism takes place anymore. This can be conveniently confirmed using glucose and ammonia quick-tests. However no such quick-tests are readily available for amino acids. To ensure the complete consumption of unlabeled amino acid substrates before feeding of the labeled variant, we determined the amino acid requirements of the CpX strains for maximum growth relative to the glucose concentration (Table 2). After reaching the stationary phase the maximal cell density, estimated by the optical density at 600 nm, was extracted (Figure 3, Figure S1). Plotting the maximal cell density against the amino acid concentration provides a convenient way to determine a realistic value for the ratio of amino acid to glucose to be used in the biomass phase (Figure 3B, Figure S1). 

It should be noted that such a procedure is not applicable to isotope label the flavin cofactor with the above-described riboflavin auxotrophic strains. Since RibM is a passive facilitator of riboflavin uptake, a quantitative uptake of riboflavin from the medium cannot be guaranteed and unlabeled flavin is expected to remain in the medium. Additionally, the turnover for flavins inside the cells is expected to be quite low and residual intracellular flavins may additionally spoil the labeling efficiency. For a quantitative labeling the labeled riboflavin must therefore be either present already at the start of the expression culture or the cells have to be washed and starved of riboflavin before induction of protein biosynthesis. Since the improved riboflavin auxotrophic strains CpXribF and CpXFMN need only about 5 µM of riboflavin for ideal vitality, only about 2 mg per liter of medium have to be applied in a typical automated high cell density fermentation experiment.

**Figure 4:**
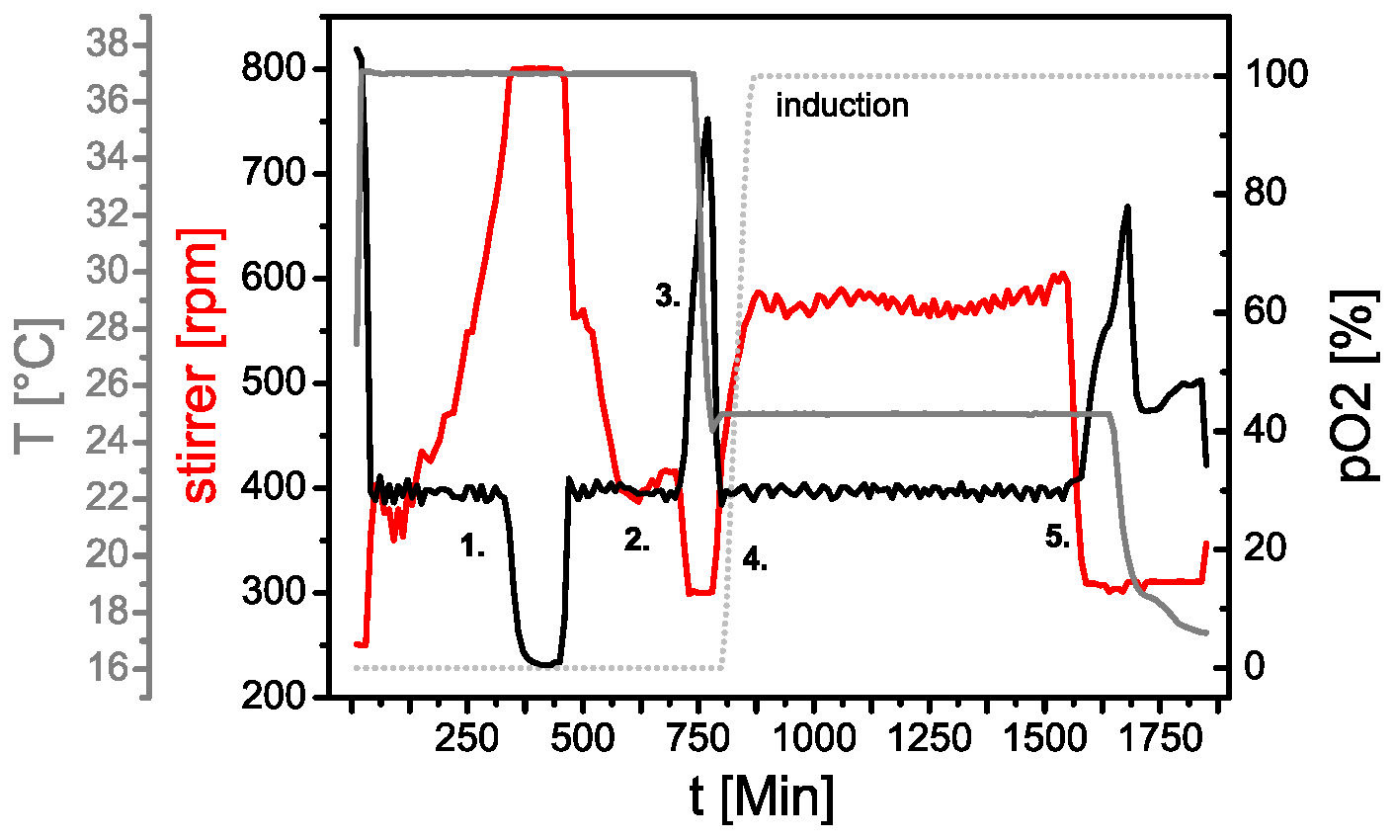
Typical profile of a high cell density fermentation experiment. In the biomass phase (1.) cells are cultivated to high cell densities. The stirrer speed (red) is gradually increased in order to keep the pO_2_ level (black) at above 30%. After the stirrer reaches its maximum speed the pO_2_ level drops to ~0%. Consumption of primary nutrients is indicated by a rise of pO_2_ (2.). Complete depletion of also secondary metabolites is recognized by a steep rise in pO_2_ (3.). This event is used to set up expression conditions, in this case by lowering the temperature from 25°C (grey). After reaching the desired temperature fresh (labeled) substrates are pumped into the culture and the pO_2_ level starts to decline again (4). At the same time protein production is induced by addition of an inducing agent (dotted grey line). After all substrates are consumed the pO_2_ level rises again and thereby indicates the end of the expression phase.

A representative fermentation profile for a selective isotope labeling experiment obtained by a scripted automatic procedure is displayed in Figure 4. The fermentation vessel was inoculated with a pre-culture of a CpX strain transformed with the expression plasmid pET28a(+)-*slr1694* and cultivated at 37°C. During the biomass production phase the oxygen consumption increases and the stirrer speed is increased accordingly to keep the pO_2_ level at above 30%. As soon as the cells have consumed all primary substrates the pO_2_ level slowly rises again, since oxidative phosphorylation is decreasing. In this phase the cells consume secondary metabolites. A sudden rise in pO_2_ then indicates that all substrates have been consumed and expression conditions are established. Here, we lower the temperature to 25°C for better folding of the expressed protein and subsequently feed labeled substrates into the culture. Protein production is induced shortly after by addition of IPTG and cultivation is maintained as long as substrates are present as indicated by the pO_2_ level. 

With this procedure we usually obtain an OD_600nm_ ~ 20 /cm of the culture medium, which corresponds to about 35-40 g of wet cell mass per liter of medium. With traditional shaking cultures (500 ml volume in a 2000 ml baffled flask) a maximum of OD_600nm_ ~ 1.5-2 /cm is reached in minimal medium. In rich (LB) medium an OD_600nm_ of about 5-8 /cm is usually obtained. The yield of soluble Slr1694 protein obtained from 1 l of fermentation culture is about 35 OD_441nm_⋅ml/cm (~70 mg). This amount is sufficient for performing extensive vibrational spectroscopic investigations by FTIR or even ultrafast transient IR absorption spectroscopy. In shaking cultures using rich medium a comparable amount of protein is obtained from ~ 4-6 liters culture, whereas in minimal medium an accordingly larger culture volume is needed. Therefore, the here described high cell density fermentation approach is clearly the method of choice for cost-effective preparation of isotope labeled proteins.

### Selective isotope labeling of amino acid side chains in Slr1694

**Figure 5:**
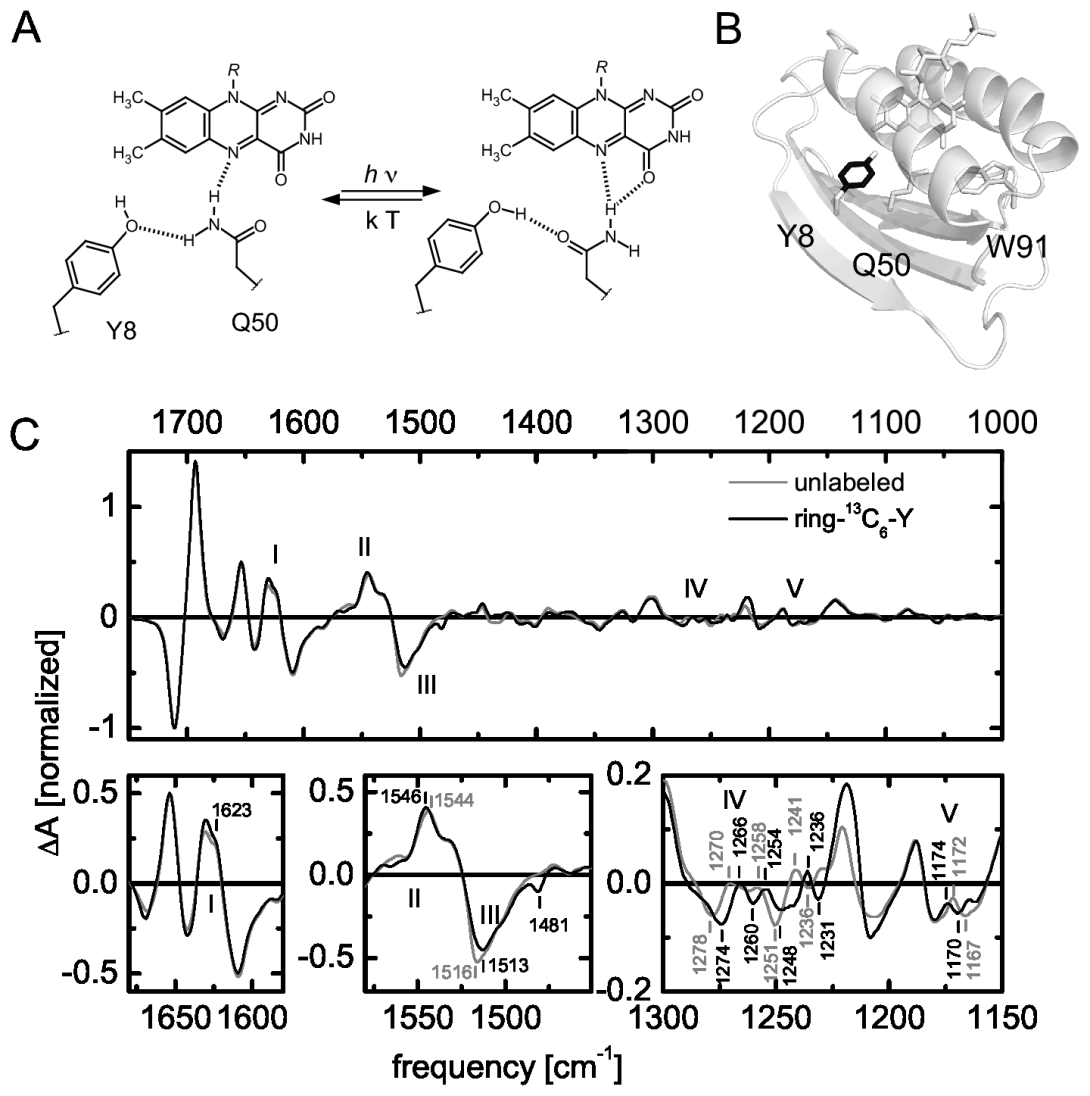
BLUF photoactivation according to the glutamine rotamer model, which accommodates hydrogen bond switching around the flavin chromophore upon illumination (A). Using the tyrosine auxotrophic expression strain CpXΔY selective isotope labeling of the phenyl ring (black) of tyrosine side chains was obtained (B). Light-minus-dark FTIR difference spectra of unlabeled Slr1694 (grey) and ring-^13^C_6_-Y labeled Slr1694 (black) in H_2_O show various isotope-induced shifts, which represent the changed hydrogen bond network around the tyrosine (C).

As an example for a successful amino acid isotope labeling experiment we compared light-minus-dark FTIR difference spectra of the unlabeled wild type BLUF photoreceptor Slr1694 (SyPixD) from *Synechocystis* *sp.* PCC 6803 with a ring-^13^C_6_-tyrosine labeled variant in H_2_O (Figure 5, Table 3). A conserved tyrosine (Y8 in Slr1694) is essential for the light-activation of BLUF photoreceptors and cannot be replaced by site-directed mutagenesis without losing photoreceptor functionality (Figure 5A,B). Therefore it is difficult to assign the spectral signature of tyrosine by vibrational difference spectroscopy on site-directed mutants. In time-resolved experiments overlapping spectral signatures may be kinetically separated and thus identified as we described earlier [13,54]. In steady-state experiments however, only isotope labeling may directly reveal the tyrosine related contributions to the spectra. It should be noted that while in ultrafast spectroscopy in the picosecond time domain only amino acids in close vicinity of the excited chromophore may be considered, steady state experiments also record changes in the periphery of the protein. Therefore, distal tyrosines may have to be considered, which are also labeled in such an approach. However, since in BLUF domains only Y8 close to the flavin is conserved, it is unlikely to observe any light-induced transitions for other tyrosine side-chains. 

**Table 3:** Tentative assignment of isotope induced shifted light-minus-dark FTIR difference signals.

**[cm^-1^**]			
**unlabeled**	**^13^C-apo**	**ring-^13^C_6_-Y**	**Assignment**
1712 (-)	1710 (-2)	n.s.	ν(C=O) flavin, mixed and/or coupled with apoprotein (this study and [10])
1693 (+)	1684 (-9)	n.s.	ν(C=O) flavin, mixed and/or strongly coupled with apoprotein (this study and [10])
1654 (+)	1617 (-37)	n.s.	Amide I (this study and [10])
1631 (+)	1591 (-40)	n.s.	see above
1544 (+)	1541 (-3)	1546 (+2)	ν(C-N) previously assigned to flavin (N5C4a / N1C10a) or amide II (1516 cm^-1^) [10]; apoprotein mixed with ν(C-C) ring tyrosine, coupled to ν(C-O) (this study and [55,67])
1530 (+,s)	1520 (-10)	n.s.	see above
1516 (-)	1500 (-16)	1513 (-3)	see above
-	1481 (-)	1481	ν(C-C) tyrosine (this study)
1345 (-)	n.s.	n.s.	flavin; predominantly assigned to ν(N3C4) (this study and [10])
1301 (+)	n.s.	n.s.	see above
1278 (-)	-	1274 (-4)	ν(C-O); δ(C-O-H) tyrosine (this study and [55])
-	-	1260	see above
1258	-	1254 (-4)	see above
1251 (-)	n.s.	1248 (-3,b)	flavin, predominatly assigned to ν(N3C4)[10]; mixed with tyrosine (this study)
1241 (+)	-	1236 (-5)	ν(C-O); δ(C-O-H) tyrosine (this study and [55])
1236 (-)	-	1231 (-5)	see above
1188 (+)	n.s.	n.s.	flavin; ρ(CH3) (this study and [10])
1179 (-)	n.s.	n.s.	see above
1172	-	1174 (+2)	ν(C-O); δ(C-O-H) tyrosine (this study and [55])
1167	-	1170 (+3)	see above

Abbreviations: s = shoulder; b = broad; n.s. = not shifted; - = unclear; ν = stretching vibration; δ = bending vibration; ρ = rocking vibration.

Previously, Takahashi and coworkers [55] identified C-O and C-O-H related structural and chemical changes in TePixD from *Thermosynechococcus elongatus* by using L-tyrosine labeled selectively at the C-4 position of the phenol ring. Here we were interested in A) if we could observe similar isotope edited spectra in the closely related protein Slr1694, B) if we could also identify light-induced changes of the aromatic ring, which were not observed in the C-4 labeled protein, and C) the preparation of sufficient amounts of protein for ultrafast time resolved IR spectroscopy. For this purpose we used L-tyrosine, labeled with ^13^C on all six atoms of the phenol ring, which was supplied during the protein production phase in a HCDF culture using the tyrosine auxotrophic strain CpXΔY as described above. With this approach we obtained ~70 mg of protein with a labeling efficiency of 90% as observed by mass spectrometry (MS) (Figure S4). Since no isotope scrambling and only ^13^C_6_ labeled peptides were found, the remaining unlabeled fraction most likely originates from unlabeled tyrosine, which has not been completely consumed in the biomass production phase. By adjusting the glucose/tyrosine ratio in this phase of the cultivation, an even higher labeling efficiency may be obtained in future experiments. In contrast, Takahashi and coworkers employed a transposon-generated tyrosine auxotrophic BL21(DE3) derivative [56] and cultivated the cells in shaking cultures using a more complex minimal medium enriched with all 20 amino acids including ^13^C-4 labeled tyrosine throughout the cultivation. While no MS data was given in their report, a high labeling efficiency is expected accordingly. Their cultivation conditions, however, would not allow for similar high yield of protein per liter as we obtain here and are therefore not suitable for our demands. 

### Light-induced structural changes of the Y8 in Slr1694

The FTIR light-minus-dark difference spectra of labeled protein, ring-^13^C_6_Y-Slr1694, were compared to unlabeled Slr1694 (Figure 5C, Table 3). By labeling of all carbon atoms of the phenol ring we observe isotope induced shifting for the C-O and C-O-H related vibrations between 1300 and 1100 cm^-1^ (IV/V) and around 1517 cm^-1^ (III) similar to the observations of Takahashi and coworkers [55]. At 1623 cm^-1^ (I) the labeled sample features a slightly broadened signal but no clear isotope induced shift. This region was previously assigned to tyrosine aromatic vibrations from ultrafast experiments [13,54]. According to studies on tyrosine in solution this frequency range corresponds to C-C ring vibrations of the phenol moiety [57]. In UV resonance Raman studies on the related AppA BLUF domain a signal at 1616 cm^-1^ was observed and assigned to the tyrosine Y8a vibration, which is also attributed to C-C phenol ring vibrations [58]. No clear light-induced differences could be assigned to this tyrosine vibration either since it strongly overlaps with the W1 vibration of tryptophan. On the ultrafast timescale the tyrosine undergoes proton coupled electron transfer, which leads to the formation of tyrosyl radical species, which naturally affects the aromatic nature of the phenyl ring. However in both light- and dark-adapted states the tyrosine is present in the same oxidation state. Effects on the C-C bond structure or the aromatic ring might be due to π-cation or π-π interactions, which were postulated in the early days of BLUF research but this idea was abandoned in the meantime [59]. 

**Figure 6:**
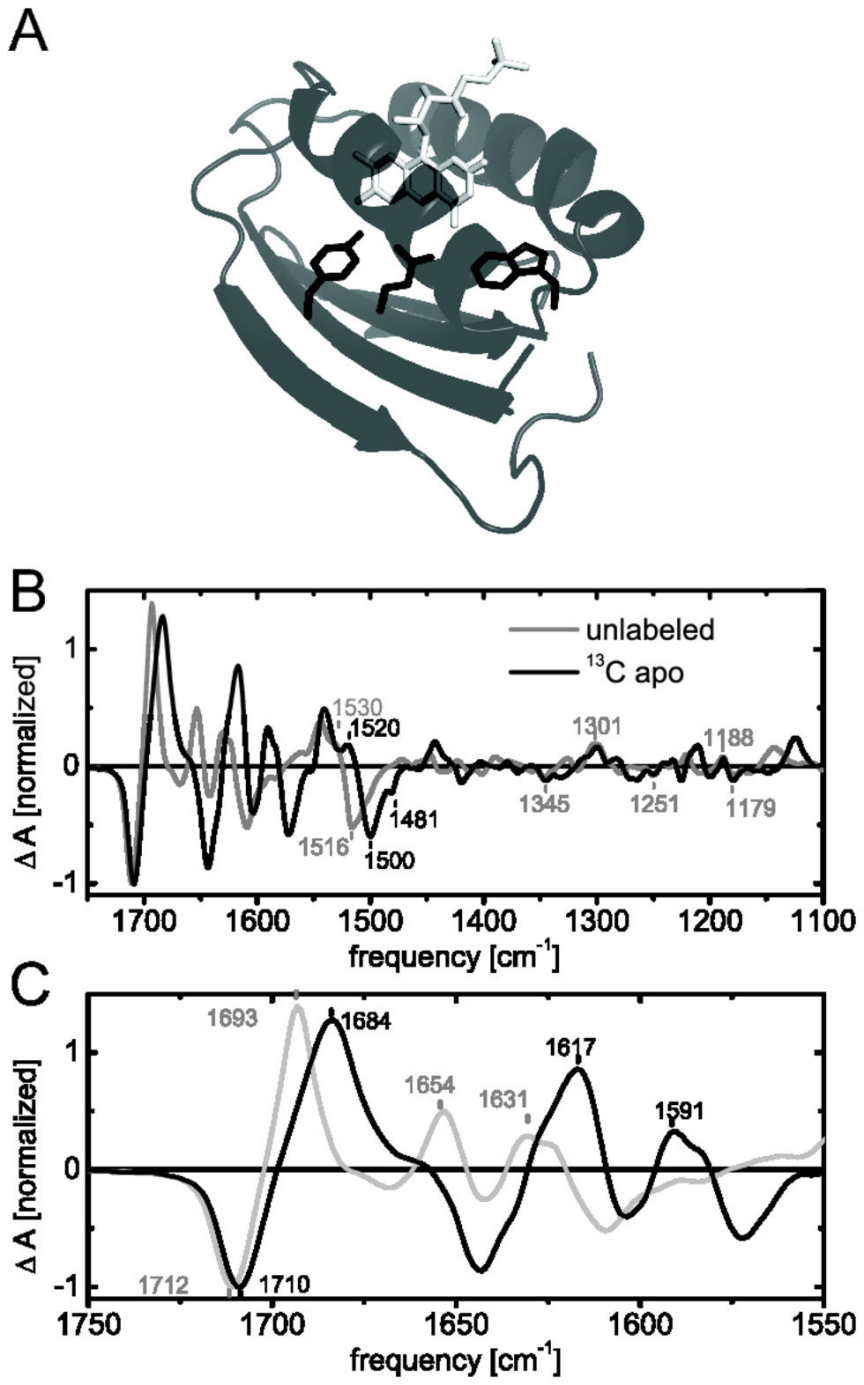
Selective unlabeling of the flavin chromophore (A, light grey) upon uniform ^13^C labeling of the protein (dark grey and black) using the riboflavin auxotrophic strain CpXribF. Light-minus-dark FTIR difference spectra of unlabeled Slr1694 (grey) and apoprotein ^13^C-labeled Slr1694 (black) in H_2_O are presented in B and C. The close-up of the amide frequency range shows a downshift of secondary structural changes as well as coupling of flavin and protein modes (C).

Further isotope-induced shifts are found at 1546 cm^-1^, where a 2 cm^-1^ upshifted (+) signal is visible in the labeled sample (II), which has not been observed before for the C-4 labeled tyrosine sample. At 1516 cm^-1^ a signal (III) is downshifted to 1513 cm^-1^ (-). Furthermore a dip at 1481 cm^-1^ appears, which is also present in the ^13^C apoprotein labeled sample (Figure 6). These signals may correspond to C-C stretching modes coupled to C-O/C-O-H modes as calculated by Takahashi and coworkers [55]. A series of signals is shifted between 1290 and 1250 cm^-1^ (IV, V), which were previously assigned to C-O and C-O-H related vibrations for a ^13^C-4 labeled tyrosine sample by the same group. The signals we observe appear more complex and at slightly different frequencies. The latter is not necessarily surprising since coupling to neighboring vibrations is expected to be different due to the different labeling pattern of the phenol ring. Additionally TePixD may have slightly different vibrational signatures than SyPixD. In total, the complete labeling of the tyrosine phenol ring clearly shows that the tyrosine predominantly changes its hydrogen bonding interactions within the BLUF domain upon illumination without any significant change in any aromatic interactions. For future experiments the ^13^C_6_-ring tyrosine isotope labeled sample will be useful to discriminate flavin and tyrosine signatures in ultrafast transient IR absorption experiments to further investigate the molecular dynamics of the proton-coupled electron transfer in BLUF domains.

### Selective unlabeling of the flavin chromophore in Slr1694

Since protein and cofactor signatures may overlap in IR absorption spectroscopy a straight-forward way to distinguish their contributions is to selectively label or unlabel the chromophore. In principle this is also possible with (pre-)resonance Raman spectroscopy, that can selectively enhance the vibrations of the chromophore, which has been successfully applied to the AppA BLUF domain before [60]. However only Raman active modes will be selectively observed and it is thereby not necessarily possible to fully cover the IR absorption spectrum of the cofactor. Nevertheless Raman spectroscopy is a powerful tool to selectively study flavin cofactors and in combination with selective isotope labeling can identify vibrational coupling of protein and flavin.

An efficient and usually less expensive way than selective labeling of amino acids or flavin is selective inverse labeling. Accordingly, an auxotrophic strain is employed to label everything except the essential amino acid or cofactor by using isotope labeled carbon and/or nitrogen sources, while the corresponding amino acid or cofactor is provided in unlabeled form. Using the same HCDF procedure as described above we employed the enhanced riboflavin auxotrophic strain CpXribF to selectively unlabel the flavin chromophore *in vivo* (Figure 6A). While labeling of the protein moiety was accomplished by fully ^13^C labeled glucose, unlabeled riboflavin was provided in the medium throughout the cultivation procedure for the production of FMN and FAD cofactors. Thus the protein part of the flavoprotein Slr1694 was uniformly labeled while the flavin cofactor remained selectively unlabeled. Such a labeling pattern has been achieved previously for the BLUF domain of the AppA protein by *in vitro* refolding of denatured protein [61]. These procedures are generally experimentally cumbersome and usually go along with loss of (labeled) protein. Additionally the *in vitro* refolded protein may have altered structural properties than the natively folded protein and not all proteins may be refolded successfully *in vitro*. 

### Light-induced structural changes of flavin and apoprotein in Slr1694

With the apoprotein uniformly ^13^C labeled and the flavin unlabeled, we can in principle assign protein signals as well as flavin signals in FTIR difference spectra directly (Figure 6, Table 3). Signals that have not been shifted compared to the unlabeled spectrum are found at 1345(-), 1301(+), 1251(-), 1188(+) and 1179(-) cm^-1^ and can therefore be unambiguously attributed to the flavin chromophore, without significant vibrational coupling to the apoprotein. These frequency ranges have been previously assigned to N3C4 stretching vibrations and CH_3_ rocking vibrations of the flavin, while the latter assignment was not completely clear before and unspecified side-chain contributions were considered as well [10]. A final molecular assignment however may only be obtained by selective isotope labeling of the corresponding atoms in the flavin chromophore. The predominantly secondary structure assigned signals in the amide I region at 1655 and 1630 cm^-1^ (+) are both downshifted, as expected, to 1617 and 1591 cm^-1^, respectively. The overall peak shape appears to be unaffected by the isotope labeling. At 1572 cm^-1^ (-) a dip is visible which probably corresponds to the 1609 cm^-1^ signal in unlabeled sample and may also originate from secondary structural changes. 

The strongest signal at 1712 cm^-1^ (-) is downshifted slightly to 1710 cm^-1^ (-), indicating that this signal predominantly consists of flavin signatures, which have been attributed to the carbonyl bond stretching vibrations of the isoalloxazine ring before. The putative corresponding positive signal at 1693 cm^-1^, which is usually attributed to the C4=O carbonyl stretching vibration receiving a novel hydrogen bond in the light-adapted state, is downshifted even stronger to 1684 cm^-1^. Therefore, a strong coupling to the apoprotein or a dominant contribution from the apoprotein is underlying these signals. Likely candidates may be glutamine or asparagine side chain amides, which characteristically absorb in this region and moreover coordinate the flavin. Furthermore a conserved glutamine residue coordinating the flavin is expected to undergo structural transitions to facilitate the signaling mechanism of BLUF domains (Figure 5A). A clear assignment of the apoprotein contributions and a mechanistic interpretation is not possible at this stage. A combination of further isotope labeling patterns, both on flavin and apoprotein, time resolved experiments and computational calculations will most likely be necessary to fully understand and visualize the role of the essential glutamine in BLUF domains. 

Further shifted signals originating from the protein alone or coupled to the flavin are found between 1450 and 1100 cm^-1^. These signals are less pronounced or offer more ambiguity concerning their assignment and are therefore not interpreted at this moment.

### Refined assignment of ν(C-N) signatures in BLUF photoactivation

At 1544 cm^-1^ (+) a signal originating from the light-adapted state is downshifted to 1541 cm^-1^ along with its shoulder from 1530 to 1520 cm^-1^. These signals have been previously assigned to CN stretching vibrations of the flavin on the basis of uniform ^13^C and ^15^N labeling of both cofactor and protein [10]. However, selective labeling shows here that these signals are clearly dominated by apoprotein signals and more precisely also tyrosine signatures (see above). The negative signal at 1517 cm^-1^ (-), which was previously assigned to CN stretching vibrations of the flavin and/or NH bending vibrations (amide II) [10], is now found at 1500 cm^-1^. The extent of the isotope induced shift of this signal is very similar to the shift observed in a uniform ^13^C labeled sample [10] and therefore rules out any contributions from the flavin. Most likely the 1517 cm^-1^ (-) and the 1530 cm^-1^ (+) shoulder reflect one vibrational signature attributed to the apoprotein, which becomes up-shifted in the light-adapted state. This frequency region is typical for indole vibrations (ν(CN), δ(CH), δ(NH)), tyrosine vibrations (ν(CC), δ(CH)), lysine vibrations (*δ*_*s*_(NH)) and amide II vibrations in general [57]. Tyrosine signatures underly these signals but only slightly account for the observed shift here (see above, Figure 5C). Lysine side chains have not been discussed in the BLUF photoactivation mechanism so far and most lysine residues in Slr1694 are found far away from flavin cofactor in the peripheral region of the protein. A likely candidate however may be a semi-conserved tryptophan residue, which is discussed as a putative signal transduction element or as a structural marker for light-induced structural changes [19,62]. Although the indol side chain in Slr1694 and BLUF domains in general is not expected to undergo drastic conformational changes [63,64], the up-shift of this signal might represent a different hydrogen bonding or electrostatic environment of the indol side chain in the light-adapted state. In resonance Raman spectroscopy this frequency region is assigned to the W3 vibration of tryptophan, which is very sensitive to the dihedral angle between indole group and Cβ atom [65] and thus the orientation of the side chain. For the related BLUF domain of AppA, UV resonance Raman spectroscopy indeed showed that the dihedral angle of the homologous tryptophan residue decreases by about 5° after illumination [58]. However since ^15^N labeled Slr1694 shows a similar shift [10], this vibration may be mainly attributed to a strengthening of CN stretching vibrations of the indol side chain in the light state. Such a strengthening could be due to a loss of a hydrogen bond, which was proposed for the dark state between the conserved glutamine and tryptophan earlier [21], but has been unfavored by recent tryptophan fluorescence and acrylic amid quenching experiments [63,66]. In an earlier FTIR study on an Slr1694 mutant lacking the corresponding tryptophan, the here discussed signal was not drastically reduced and therefore mainly attributed to CN stretching and NH deformation vibrations of the protein backbone [67]. The contribution of tryptophan to this signal therefore needs to be confirmed by selective isotope labeling of tryptophan residues in future experiments. 

### “Reactivity labeling” of Slr1694

The isotope labeling approach described here can also be used to selectively tune the chemistry of protein and cofactor as an alternative to site-directed mutagenesis. We previously reported that a riboflavin auxotrophic strain is suitable to reconstitute flavin analogs into flavoproteins directly *in vivo* without the need to first unfold the protein to release the cofactor and subsequently refold it in the presence of the flavin analog [37]. In case of the reported roseoflavin reconstituted LOV and BLUF photoreceptor proteins, we were able to investigate cofactor induced reactivity changes by absorption and fluorescence methods [45,68]. Of course this approach is limited to flavin analogs that are translocated by RibM, but the spectrum of transported flavin analogs is expected to be broad. 5-deaza-riboflavin for example is also transported by RibM and was used to reconstitute the *Chlamydomonas reinhardtii* phototropin LOV1 domain *in vivo* (Mansurova & Mathes, unpublished observation).

This methodology is also suitable to introduce chemically modified amino acids into the protein of choice. Depending on the nature of the chemical modification we are able to fine tune the reactivity of a given amino acid side chain, which may not always be possible with the limited spectrum of the 20 canonical amino acid side chains available in nature. In BLUF domains for example the tyrosine electron donor cannot be functionally replaced by any other amino acid. Furthermore also neighboring amino acid side chains, which in principle should influence the chemistry of the tyrosine, cannot be functionally replaced either. However, we managed to prepare a functional BLUF domain containing fluorinated tyrosines [15]. With this approach we were able to fine tune the redox potential of the primary photoinduced electron transfer step in this protein in such a way, that a previously occluded photocycle intermediate became observable. One has to keep in mind though, that this only works for slightly modified amino acid analogs that are recognized by the corresponding aminoacyl-tRNA-synthetases.

## Conclusions and Perspective

Selective isotope or reactivity labeling procedures are sophisticated techniques to understand biological reactions in general and are crucial to assign and finally interpret vibrational spectroscopic data as demonstrated here for BLUF photoreceptors. Although we managed to resolve some ambiguous assignments and propose new ideas for the differences between dark and light-adapted states of BLUF photoreceptors, clearly more selective isotope labeling patterns will be needed to fully resolve the molecular structural transitions in BLUF signaling. We show here that such an approach can be set up efficiently with widely established molecular biological methods and microbial fermentation techniques to meet the high sample demands of e.g. time resolved vibrational spectroscopy. The set of auxotrophic strains presented here will be used to elucidate flavoprotein mechanisms by vibrational and NMR spectroscopy and serve as a basis for further (multi-) auxotrophic strains to be engineered in the future.

## Supporting Information

Figure S1 **Growth characteristics of CpXΔH (**A**), CpXΔW (**B**), CpXΔN(**C**) and CpXΔC (**E**).**
(EPS)

Figure S2 Comparison of growth characteristics of CpXΔQ, CpXFΔQ, CpXΔQ* and CpXFΔQ* (A). In (B) the growth characteristics of CpXΔW and CpXFΔW are displayed.
(EPS)

Figure S3 **Estimation of signal to noise ratio of the here presented FTIR difference spectra of unlabeled (**A**), ring-^13^C_6_-Y labeled (**B**) and ^13^C apo protein labeled Slr1694 (**C**), all prepared in H_2_O.** The upper panel displays the average of 10 x 100 scans of light-minus-dark (red) and dark-minus-dark spectra (black). The corresponding standard variation is given in the lower panels.
(EPS)

Figure S4 **MALDI mass spectrometry of ring-^13^C_6_ Y labeled Slr1694 after trypsin digestion.** A – signals originating from the peptide ^33^GSEFMSLYR^41^ at m/z 1089.507 in the unlabeled and m/z 1095.527 in the labeled form; B - signals originating from the peptide ^42^LIYSSQGIPNLQPQDLK^58^ at m/z 1914.038 in the unlabeled and m/z 1920.059 in the labeled form. The +1 and +2 m/z signals correspond to the natural abundance of ^13^C. Note: the numbering of the peptides is derived from the expressed construct from pET28a(+)-*slr1694*. The essential tyrosine (Y8) is found in the second peptide (B).
(EPS)
